# Latitudinal clines in gene expression and *cis*-regulatory element variation in *Drosophila melanogaster*

**DOI:** 10.1186/s12864-016-3333-7

**Published:** 2016-11-28

**Authors:** Punita Juneja, Andrew Quinn, Francis M. Jiggins

**Affiliations:** Department of Genetics, University of Cambridge, Cambridge, CB2 3EH UK

**Keywords:** Drosophila, Allele-specific expression, Cline

## Abstract

**Background:**

Organisms can rapidly adapt to their environment when colonizing a new habitat, and this could occur by changing protein sequences or by altering patterns of gene expression. The importance of gene expression in driving local adaptation is increasingly being appreciated, and *cis*-regulatory elements (CREs), which control and modify the expression of the nearby genes, are predicted to play an important role. Here we investigate genetic variation in gene expression in immune-challenged *Drosophila melanogaster* from temperate and tropical or sub-tropical populations in Australia and United States.

**Results:**

We find parallel latitudinal changes in gene expression, with genes involved in immunity, insecticide resistance, reproduction, and the response to the environment being especially likely to differ between latitudes. By measuring allele-specific gene expression (ASE), we show that *cis*-regulatory variation also shows parallel latitudinal differences between the two continents and contributes to the latitudinal differences in gene expression.

**Conclusions:**

Both Australia and United States were relatively recently colonized by *D. melanogaster*, and it was recently shown that introductions of both African and European flies occurred, with African genotypes contributing disproportionately to tropical populations. Therefore, both the demographic history of the populations and local adaptation may be causing the patterns that we see.

**Electronic supplementary material:**

The online version of this article (doi:10.1186/s12864-016-3333-7) contains supplementary material, which is available to authorized users.

## Background

It is common for traits to vary across the geographical range of a species, and this is frequently linked to adaptation to the local environment. Latitudinal clines, which correlate with environmental conditions such as temperature, UV radiation, humidity, and pathogen abundance, present an ideal system for studying local adaptation [1–3]. For example, it is common to find across many species that body size is smaller nearer the tropics, and this is associated with increases in environmental temperature [4]. Direct evidence that such clines are driven by adaptation has come from reciprocal transplant experiments showing reduced fitness at different latitudes [5] and from an understanding of the function of the phenotypic differences in different environments. More indirectly, evidence for adaptation comes from the observation that the same latitudinal patterns evolve repeatedly either in nature [6] or in laboratory conditions that mimic latitudinal differences such as temperature [7].

In *Drosophila melanogaster* there is clinal variation of phenotypic traits along the east coasts of Australia and the United States [8, 9]. Despite these areas having only been colonized in the past few hundred years, in many cases parallel clines occur on the two continents. Typically tropical populations have smaller bodies, greater resistance to high temperature, reduced tolerance of low temperature and lack the ability to enter reproductive diapause in the winter [8, 9]. The clear link of these phenotypes to changes in climate, together with the fact that they occur across multiple continents, suggests that these clines are a product of local adaptation that is maintained by spatially varying selection [10]. In some cases the genetic basis if these phenotypic differences is known, and the polymorphism has been found to vary clinally. For example, a single amino acid change in the *couch potato* gene is associated with reproductive diapause and shows clinal variation that closely mirrors changes in the trait [11].

The advent of new sequencing and genotyping technologies has allowed the search for clinal variation to be extended to the entire *D. melanogaster* genome [1, 12]. This resulted in large numbers of clinally varying SNPs being discovered, and the finding that parallel clines were commonly found in the US and Australia suggested that many different genes across the genome might be involved in local adaptation [13]. However, recent analyses suggest that clinal patterns may instead result from demographic processes [14, 15]. Both Australian and US populations of *D. melanogaster* have been founded from both African and European populations, with African flies contributing primarily to populations nearest the tropics on both continents [14, 15]. Therefore, much of the clinal variation in genetic markers may simply reflect admixture between European and African flies and not local adaptation, and parallel clines of genetic variants alone cannot be taken as evidence of local adaptation [14, 15].

A small number of studies have investigated geographical variation in gene expression in *D. melanogaster*. When populations of *D. melanogaster* from Maine USA and Panama were compared, numerous genes were found to be differentially expressed [16]. *D. simulans* populations collected from the same locations also showed differences in gene expression, and these frequently involved the same genes changing in expression in the same direction [16]. The observation that the same patterns have arisen in parallel in the two species strongly suggests that these differences in gene expression are in part driven by spatially varying selection pressures [16]. In Australia transcriptional differences have been identified using microarrays between northern and southern Australia [17, 18]. When flies from tropical and temperate regions of Australia are reared at both hot and cold temperatures, there is an excess of genes that are downregulated at the temperature that is most unlike the flies’ natural environment [18]. The genes showing these patterns were clustered in small groups, which suggests some of the changes may be due to chromatin regulation [18]. This observation that plasticity in gene expression is linked to environmental variation suggests that this variation is in part an adaptation to maintain correct levels of gene expression when flies colonized regions with different temperatures [18].

Selection on gene expression has long been argued to be the major source of evolutionary change [19], and therefore may be important to local adaptation. Local adaptation via changes in gene expression has recently been demonstrated to be more common than by amino acid substitutions in humans [3]. Modification of expression can occur via *cis-*regulatory elements (CREs), which are physically linked to the genes whose expression they control, or *trans*-acting elements, which are physically distant. CREs tend to influence one or a few gene targets, often in specific tissues or at specific times, whereas *trans* factors can control the expression of many genes. This means that the modification of CREs during evolution may result in fewer pleiotropic changes than changes to *trans* acting factors [20]. Therefore, it is expected that CREs are an important source of local adaptation, as they allow the fine-tuned alteration of gene expression with fewer associated fitness costs [20].

The effects of *cis* and *trans*-acting polymorphisms can be distinguished by measuring the relative expression of the two alleles of a gene within a single individual [21, 22]. Polymorphisms acting in *trans* are expected to alter the expression of the two alleles equally, while *cis*-acting polymorphisms will result in the expression of the alleles on the two chromosomes differing. This allele-specific expression (ASE) can be detected by measuring the relative abundance of SNPs in RNAseq reads, with deviations from a 50:50 ratio indicating ASE [23–27]. Such analyses must be undertaken with care, as RNAseq reads are less likely to map to a reference genome when SNPs cause mismatches to the reference genome, generating false signals of ASE [28]. These problems have been overcome in a variety of ways, allowing *cis-*regulatory variation to be studied on a genome-wide scale.

**Fig. 1:**
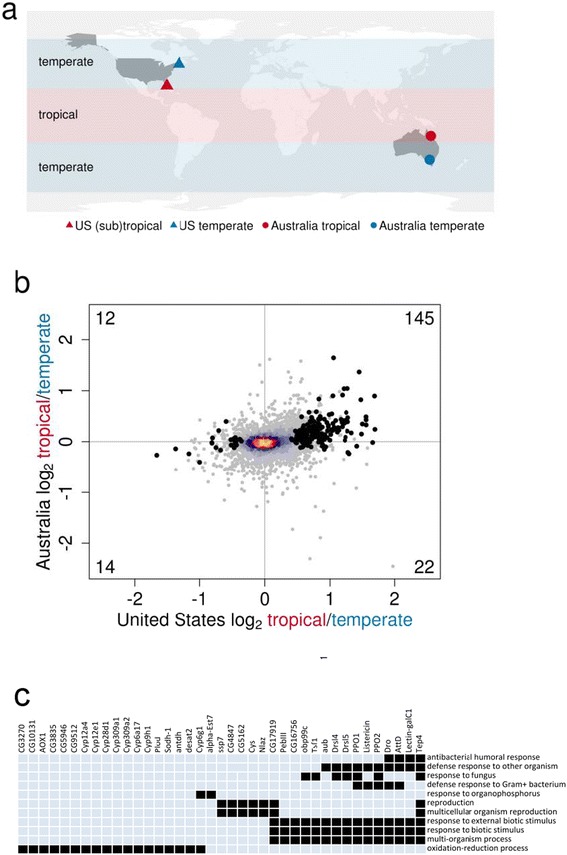
Parallel differences in total gene expression between temperate and tropical populations in Australia and the United States. **a** Temperate (*blue*) and tropical or sub-tropical (*red*) populations were sampled from Victoria and Queensland in Australia (*circles*) and Maine and Florida in the United States (*triangles*). **b** Mean per gene log_2_ fold expression differences between tropical and temperate populations were estimated separately for each continent. Genes that are significantly differentially expressed on either continent (FDR < 0.20) are shown in *black*, and the number of significant points in each quadrant is shown in the corner. Warmer colours indicate a greater density of superimposed points. **c** GO term enrichment was performed on genes that showed evidence for parallel differences between latitudes on both continents (FDR < 0.20 on one continent and the same direction of change in the other continent, *N* = 139 genes). Significant biological process categories are shown horizontally and genes in those categories vertically (*p* < 0.001; indicated by *black squares*). *Red in the bottom bar* indicates genes upregulated in tropical populations and *blue* indicates genes upregulated in temperate populations. The map was created using the R package *maps*

To study latitudinal variation in gene expression, we used 52 isofemale lines from two Australian and two United States populations, chosen to represent temperate and tropical or sub-tropical environments from each continent (Fig. 1a; 11–14 lines per population with 4 of these lines per population having biological replicates). We genetically cloned a haploid genome from each line, and then crossed these genomes to the strain of *D. melanogaster* that was sequenced to produce the standard reference genome [29] (see Additional file 1). Local adaptation to pathogens is common in natural populations [30, 31], and in *Drosophila* latitudinally differentiated genomic regions are enriched for genes involved in the immune response [1, 2, 12] and some tropical populations have higher resistance to infection than temperate populations [32]. To increase the amount of sequence data from immunity genes, we inoculated the progeny of the cross with a cocktail of heat-killed *Micrococcus luteus* (gram-positive) and *Escherichia coli* (gram-negative) bacteria to upregulate genes involved in the immune response. These bacteria were chosen as they activate the Toll and IMD pathways, which are the two main immune signaling pathways of *Drosophila* [33]. We then measured both total gene expression and allele-specific gene expression (ASE) using RNA-seq.

## Results

### Parallel differences in gene expression between tropical and temperate populations on different continents

We find evidence of reciprocal latitudinal differences in gene expression in the United States and Australia (Fig. 1b). We tested for latitudinal differences in expression by looking for an excess of genes that had significantly different expression levels between latitudes in at least one continent and that tended towards the same direction on both continents (i.e. higher expression in both temperate populations or in both tropical populations). In the United States 193 of 8717 genes were differentially expressed between the temperate and sub-tropical populations with a genome-wide false discovery rate of 20% (59 of these genes had a false discovery rate of 5%). Of these, in 83% of cases (159 of 192 genes tested in both populations) the direction of expression was the same between the temperate and tropical populations in Australia, reflecting an excess of genes with the same direction of change on both continents (Fig. 1b; 95% binomial confidence interval: 77–88%). If only the 59 genes with a false discovery rate of 5% in the US are considered, then in 90% of cases the direction of expression was the same between the temperate and tropical populations in Australia (95% binomial confidence interval: 80–95%). Furthermore, the relative expression of genes in our temperate and sub-tropical populations in the United States was positively correlated with published microarray data comparing Europe with Africa [34] (Spearman’s rank correlation: ρ = 0.10, *p* = 0.7 × 10^−8^). This pattern was largely driven by genes upregulated in tropical compared to temperate populations. When the reciprocal analysis was done, we found far fewer genes were significantly differentially expressed between latitudes in Australia (2 of 8739).

The genes with parallel latitudinal differences in expression are enriched for certain functional categories (Fig. 1c). This includes the immune response, including antimicrobial peptides (*Dro*, *AttD*, *Drsl4*, *Drsl5*, and *Listericin*), a thioester-containing protein (*Tep4*), two prophenoloxidases (*PPO1* and *PPO2*), and two lectins (*Lectin-galC1*, *lectin-33A*) (Fig. 1c, Additional file 1: Table S1). These immunity genes are all more highly expressed in the tropics. We also find higher expression in tropical populations of genes involved in pesticide resistance (*Cyp6g1*, *alpha-Est7*), melanisation and pigmentation (*yellow-b*, *yellow-e*, *Rh3*), oxidation-reduction reactions (including several cytochrome P450 genes that can affect susceptibility to toxins, *Sodh-1*, *antdh*, *desat2*), metabolism (*Mal-A1*, *Mal-A2*, *Mal-A4*, *Mal-A6*, *Mal-B1*), cuticle formation (*Cpr60D*, *Cpr65Au*, *Cpr49Ab*), and reproduction (*Cys*, *Obp19d*, *PebIII*, *NLaz*, *ssp7*).

### Variation in *cis*-regulatory elements alters gene expression

Gene expression can be affected by variation in *cis*-regulatory elements, which will affect expression in an allele-specific manner, or *trans*-acting factors, which will affect both alleles equally [35]. Because *cis-*regulatory variation causes allele-specific differences in expression (ASE), it can be detected by testing whether RNA-seq reads originating from the two alleles deviate from the expected 1:1 ratio [35]. For each gene, we took the sequence reads that contained a SNP and counted the numbers that came from the reference genome and fly line sampled from the wild [28]. As we have biological replicates of 12-16 genotypes for each gene we analysed (4 genotypes in each population had a biological replicate), we can detect heterogeneity in ASE using a generalized linear mixed model that accounts for any non-genetic variation in the ratio of reads from the two alleles, such as might arise during the sequencing process. Of the 3626 genes that met our coverage requirements, 660 (18%) had significant ASE with a genome-wide false discovery rate of 20% (Additional file 1: Table S2).

**Fig. 2:**
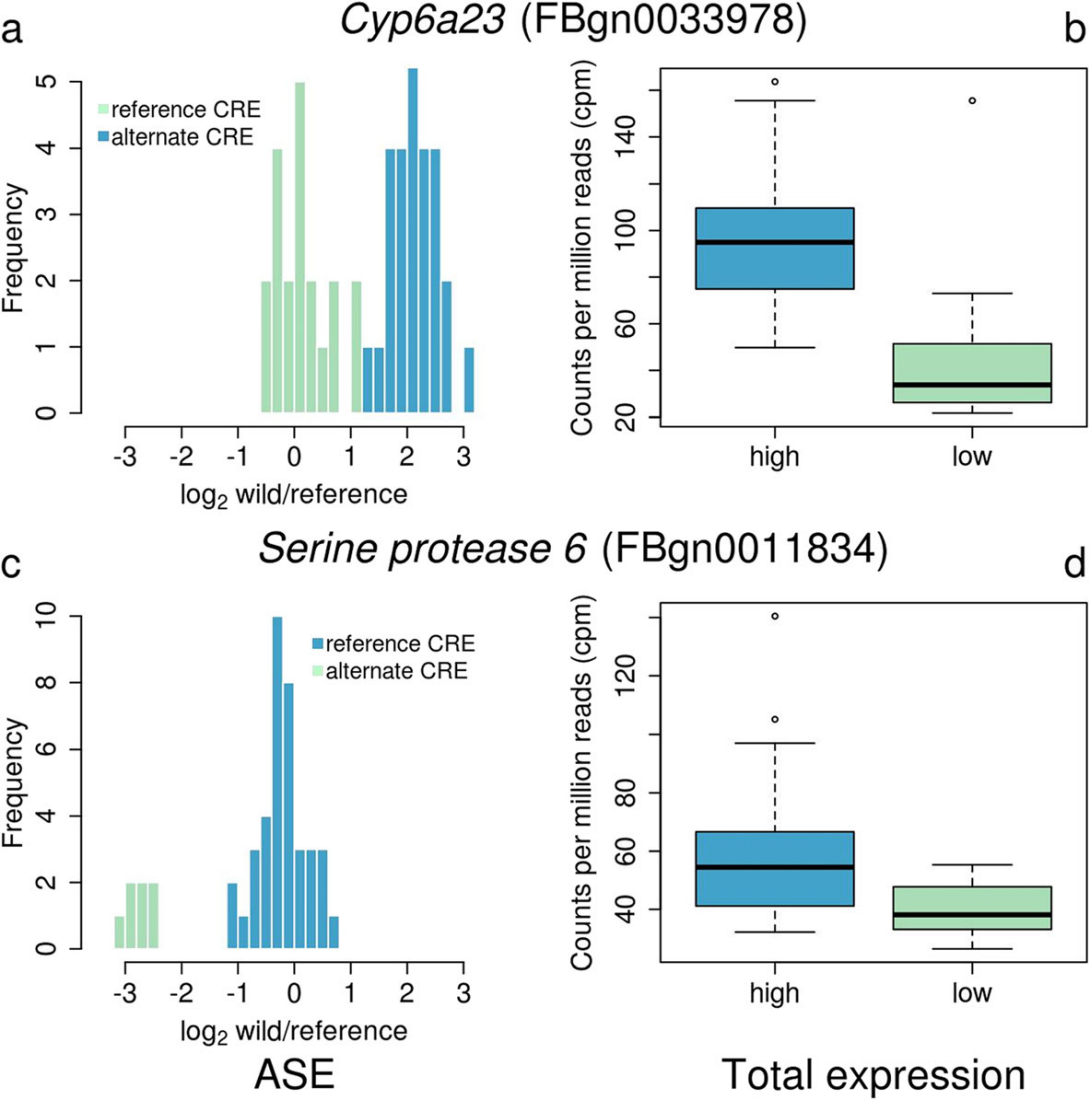
Identifying high and low expression alleles. Two genes with allele specific expression (ASE) are shown. ASE was detected as significant variation in the ratio of sequence reads from wild alleles sampled from natural populations and the reference allele across the different fly genotypes. The lines were divided into those with high and low expression alleles using a grouping approach with maximum likelihood (panels **a** and **c** in *blue*, high and *green*, low). The group with the mean log_2_ ratio closest to 0 was assumed to have the *cis*-regulatory element (CRE) allele found in the reference genome. Note that in **a** the reference genome has the low expression allele (mean log_2_ ratio of alternate group > 0), and in **c** it has the high expression allele (mean log_2_ ratio of alternate group < 0). Panels **b** and **d** show the total expression of lines carrying the high and low expression alleles

**Fig. 3:**
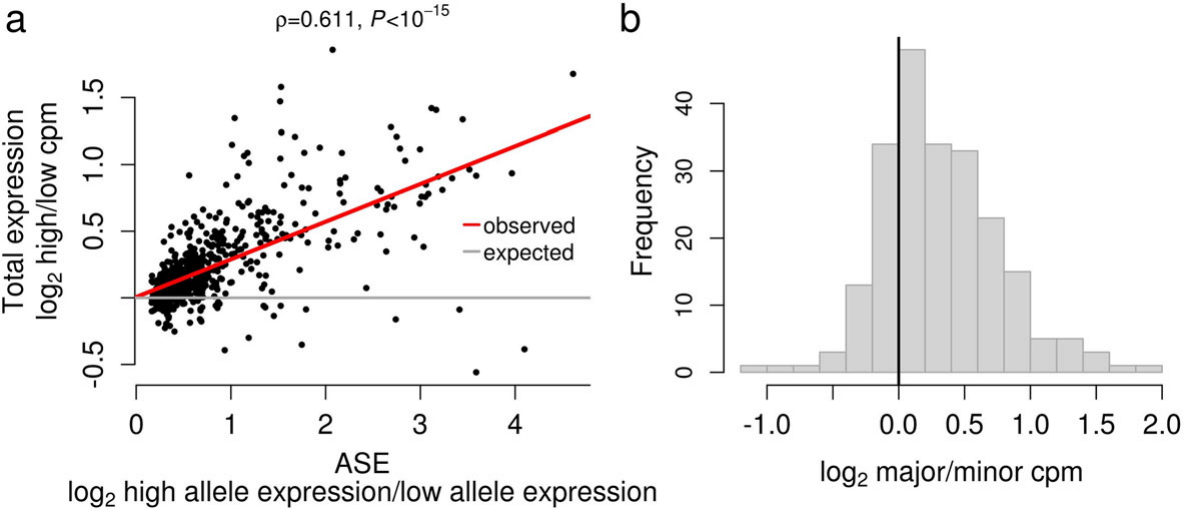
Effect on total expression and population frequency of *cis-*regulatory polymorphisms. **a** ASE, measured as the imbalance in expression of high versus low alleles, has a phenotypic effect on total expression. The relative total expression of each gene in lines containing the high versus low ASE alleles was estimated as the log_2_ ratio of the mean cpm (counts per million) of the high and low allele groups. ASE was estimated as the difference between the high and low expression groups in the mean log_2_ wild-allele-read-count/reference-allele-read-count ratio (see Fig. 2). **b** The major ASE allele tends to have higher total expression than the minor allele (mean log_2_ major/minor cpm = 0.311, *t*-test μ ≠ 0 *P* < 10^−15^). In **b** only genes where the minor allele had a frequency of less than 30% are analysed. In all cases, genes were considered to have ASE at an FDR < 0.20

Using our estimates of ASE, we divided the alleles of each gene into high and low expression groups (estimated from the ratio of reads from the reference genome and the fly line sampled from the wild; Fig. 2, Additional file 1). As expected, we found a strong correlation between ASE and total gene expression, which was measured separately using the total number of reads mapping to each gene [36] (Fig. 3a). In 85% of cases, lines carrying the high expression allele had higher total gene expression than lines carrying the low expression allele (Figs. 2 and 3a). We also found that the allele that was more frequent in the population (the major allele) usually had higher expression than the less frequent minor allele (Fig. 3b; combining data from all populations). Of the 221 genes with a minor allele frequency below 30% in the population, 76% (168 genes) had higher expression of the major allele. This may suggest the presence of low frequency slightly deleterious mutations reducing gene expression, as might be expected if new mutations reduce gene expression more often than they increase it.

### Parallel differences in *cis*-regulatory elements between tropical and temperate populations on different continents

**Fig. 4:**
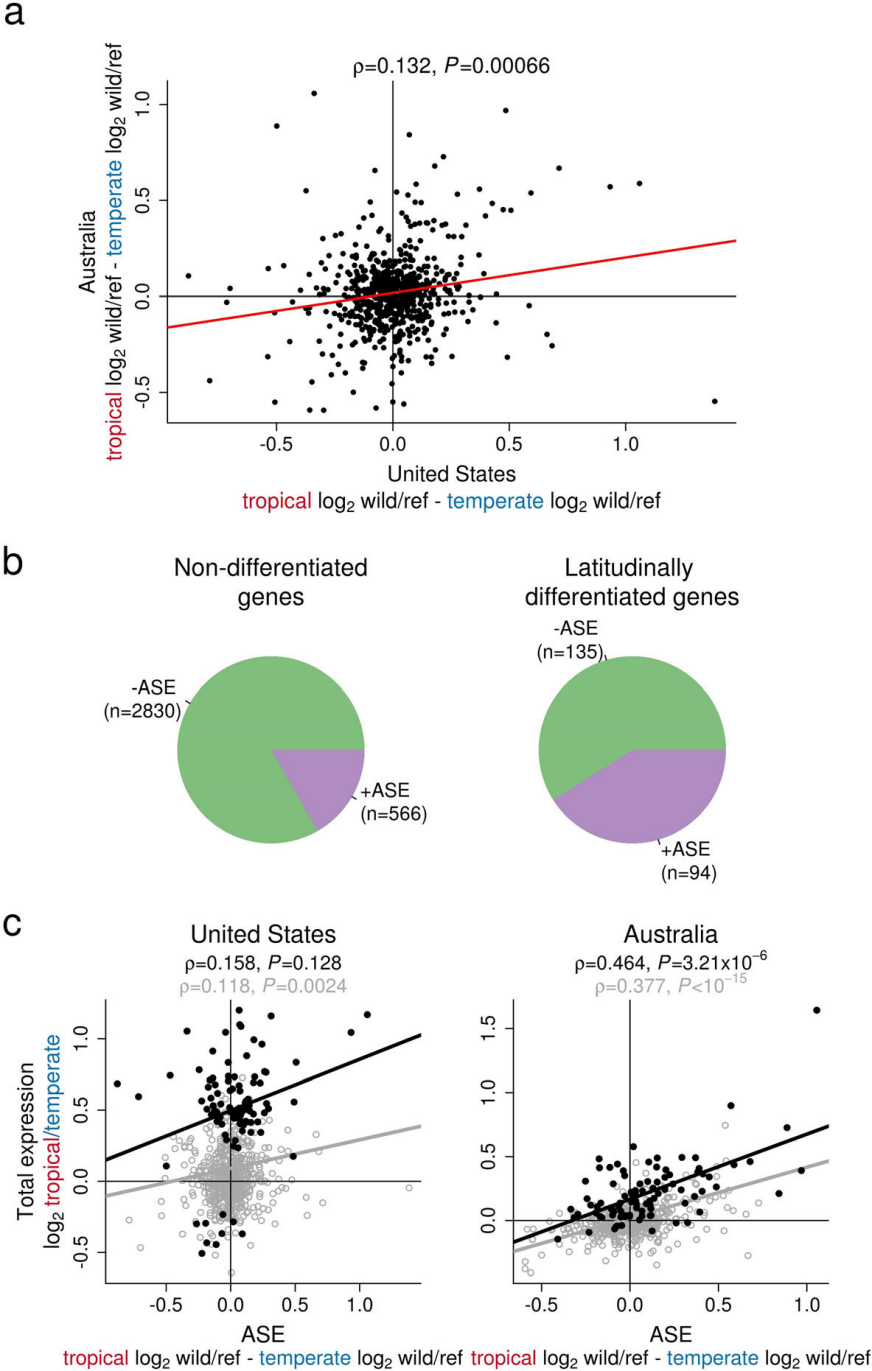
Latitudinal correlations in allele-specific expression. **a** The difference in ASE between tropical and temperate populations is significantly correlated between Australia and the United States (Spearman’s rank correlation; all genes with significant ASE are shown). **b** Genes with latitudinal differentiation in total gene expression are enriched for ASE, with 41% of these genes showing evidence for ASE compared with 17% of genes without latitudinal differentiation (Fisher’s Exact Test *P* < 10^−15^). **c** ASE differences between latitudes are correlated with total expression differences between latitudes for genes with significant ASE (*gray open circles*) and for the subset of those genes that also had significant latitudinal differentiation in total gene expression (*black solid circles*, significant only in Australia). Genes were considered to have significant ASE at FDR < 0.20, and were considered to have latitudinal differentiation in total gene expression if the latitudes significantly differed at a *P* < 0.05 on one or both continents and if the direction of change was the same on both continents

As was the case for total expression, we found reciprocal latitudinal differences in ASE in the United States and Australia (Spearman’s rank correlation ρ = 0.132, *P* = 6.6 × 10^−4^; Fig. 4a). The genes that show parallel patterns of latitudinal ASE variation include those involved in the immune response, cold acclimation, circadian rhythm, starvation response, lipid uptake and UV radiation response (Additional file 1: Figure S1A). For example, we find that in *(6-4) photolyase*, which repairs UV-induced DNA damage [37], a CRE associated with high expression has higher frequencies in both tropical populations (Additional file 1: Figure S1B).

The reciprocal latitudinal differences in total gene expression in Australia and the United States can in part be explained by CRE variation. We found that genes were twice as likely to have significant ASE if they also had parallel latitudinal differences in total gene expression (Fig. 4b). This enrichment was not driven by differences in power between genes with and without latitudinal differences in total expression, as down-sampling to correct for this yielded similar results (Additional file 1: Figure S2). Across all genes with ASE, the difference between tropical and temperate populations in ASE and total expression tends to go in the same direction in both Australia (Fig. 4c, Spearman’s rank correlation ρ = 0.377, *P* < 10^−15^) and the United States (Fig. 4c, Spearman’s rank correlation ρ = 0.118, *P* = 0.0024). This pattern holds if the analysis is restricted to just those genes with significant latitudinal differences in total gene expression, although it is only significant in Australia (Fig. 4c, black solid circles; Spearman’s rank correlation US: ρ = 0.158, *P* = 0.128; Australia: ρ = 0.464, *P* = 3.21 × 10^−6^). Together, these results demonstrate that the frequency of CRE polymorphisms vary in response to latitude and in similar ways across the two continents.

Our method for detecting ASE [28] aims to minimize the effects of mapping bias, which occurs when alleles more similar to the reference genome are more likely to map, leading to false detection of ASE. Briefly, we minimized this bias by mapping first to the reference genome and then to a genome updated to include SNPs found in the first round of mapping [28]. In addition, we only used SNPs in a high quality SNP database [38] for measuring ASE. Because all our fly lines were crossed to the reference genome strain we can detect the effect of mapping biases [28]. We found that the average proportion of reads from the reference genome allele was 50.1% and 37 of 52 lines have above 50% reference alleles, suggesting a small bias remains. However, we could rule out mapping bias as a confounding factor by repeating all our analyses separately using just genes that were biased towards or away from the reference genome (>55% or <45% reads with the reference allele) (Additional file 1: Figures S3 and S4). Inversions, which are thought to suppress recombination between locally adapted alleles, vary in frequency along latitudes in *D. melanogaster*, and the *In(3R)P* inversion is thought to carry genes associated with adaptation to latitude [1, 2, 39]. We find no evidence that genes that are latitudinally differentiated in total expression are clustered within inversions or chromosome arms (Additional file 1: Table S3), nor that latitudinal correlations in ASE are driven by particular regions of the genome (Additional file 1: Table S4).

## Discussion

We have found that changes in gene expression and allele specific expression in *Drosophila* from recently colonized temperate and tropical or sub-tropical regions in the northern and southern hemispheres are correlated. A key question is whether these differences reflect demography or local adaptation. If these differences were already present in ancestral African and European populations, unequal colonization by these founding populations may have contributed to or produced the observed differences in Australia and the United States populations. Indeed, tropical populations in both Australia and the United States have a greater proportion of African ancestry [14]. However, it is also plausible that selection has been important in establishing some of the parallel clines, as it is known that within Australia gene expression in tropical and temperate populations respond differently to changes in a temperature in a way that suggests there has been local adaptation to maintain gene expression at different temperatures. Additional evidence that selection can play a role in establishing latitudinal clines in gene expression comes from the observation that parallel clines exist in *D. melanogaster* and *D. simulans* [16]. Furthermore, it is clear that there is parallel local adaptation to latitude in phenotypic traits on both continents and it is likely that gene expression differences contribute to these differences.

The functional categories of genes that have parallel clines in expression are compatible with these differences arising due to local adaptation, regardless of whether selection was acting in the ancestral European and African populations, or in Australia and the United States. For example, immune response genes are more highly expressed in the tropics, which is consistent with previous observations of higher resistance to infection in some tropical *Drosophila* populations [32] and increased pathogen diversity closer to the equator in other species [40, 41]. Similarly, we found differences in genes involved in pigmentation, the repair of UV damage to DNA, detoxification pathways and reproduction. It is possible that these contribute to latitudinal clines in traits like reproductive rate and pigmentation that are thought to be local adaptations. Similar patterns are also seen in humans, where a recent meta-analysis has shown that expression of genes involved in the immune response, UV radiation response, and diabetes pathways are correlated with latitude [3].

Unexpectedly, we found far greater latitudinal differences in total gene expression in the US than in Australia. This difference may be explained by the larger climatic difference between the populations in the United States (Florida versus Maine) as compared with Australia (Queensland versus Victoria) resulting in larger differences in gene expression. Alternatively, it could be the result of different colonization processes on the two continents – for example Australia and the United States may have been colonized from different populations.

## Conclusions

We have found extensive latitudinal differences in gene expression which are correlated with *cis*-regulatory differences between the same populations. Many of the genes involved have functions that may be important in adaptation to local environmental conditions. A challenge for the future is to disentangle any effects of selection from demography, and to determine whether any selection has acted in recently colonized new world populations or the old world populations from where these came. One approach is to show the adaptive significance of individual genes we identified – for example, does increased expression of *(6-4) photolyase* result in reduced DNA damage in tropical populations? Alternatively, genome-wide approaches could be taken such as examining changes in ASE at different temperatures, showing parallel changes in situations where population admixture is not a problem (eg across seasons, with altitude or across species), or attempting to disentangle selection and demography analytically.

## Methods

### Fly lines and crosses

*Drosophila melanogaster* isofemale lines from Bowdoinham, Maine (44.0°N, 69.9°W) and Homestead, Florida (25.5°N, 80.5°W) comprised our temperate and sub-tropical US populations respectively, while isofemale lines from Innisfail (17.6°S, 146.0°E) and Melbourne (37.8°S, 145.0°E) comprised our temperate and tropical Australian populations respectively.

We genetically cloned a haploid genome from each isofemale line and crossed this genome to a standard isogenic fly line to obtain the flies in which we measured gene expression. In the first generation (G0; see below), we placed 2–5 virgin females per isofemale line into a large vial with two males from a T(2;3)*CyO*-*TM6* balancer stock in which the second and third chromosome co-segregate. From the resulting progeny, we collected a single male per line that exhibited the balancer phenotype and crossed it with 14 virgins from the *y; cn bw sp* isogenic strain whose genome is the standard *D. melanogaster* reference genome sequence [29] (G1; see below). We transferred these adults into a new bottle after two days and then removed the flies after 4 days to create two biological replicates for each line (R1 and R2). Three days after the first emergence for each replicate, we put flies into a new bottle and collected females not exhibiting the balancer phenotype. These flies have one set of chromosomes from the reference strain and one set from the isofemale line used in the first cross. All crosses were carried out on standard cornmeal fly media, and we randomized the positions of vials and bottles throughout the crossing period and subsequent experiment. In this design, the 4^th^ chromosome was not genetically cloned and the small number of genes on this chromosome have been excluded from the analysis. All flies were reared at 25 °C on a 12 h light-dark cycle at 70% relative humidity.

$$ \mathrm{G}0\kern1.5em \mathrm{Female}\kern0.5em {x}^{+};\ {2}^{+}; {3}^{+} \times \frac{T\left(2;3\right)CyO-TM6}{pr\ cn;\ mwh\ ry\left[506\right]\ e}\kern0.5em \mathrm{Male} $$

$$ \mathrm{G}1\kern1.5em \mathrm{Male}\kern0.5em \frac{y}{x^{+}};\ \frac{T\left(2;3\right)CyO-TM6}{2^{+};{3}^{+}} \times {x}^{ref};\ {2}^{ref};\ {3}^{ref}\mathrm{Female}\ \left(\mathrm{reference}\right) $$

$$ \mathrm{G}2\kern1em \frac{x^{+}}{x^{ref}};\ \frac{2^{+}}{2^{ref}};\ \frac{3^{+}}{3^{ref}} $$

#### *Drosophila* crosses

Chromosomes labeled with + represent those from experimental lines, while those with $ref$ are from the reference strain.

### Immune challenge via injection

Local adaptation to pathogens is common in natural populations [30, 31], and immunity genes in *Drosophila* are more likely to show clinal patterns of variation than other genes [1, 2, 12]. Furthermore, phenotypic studies have found latitudinal differences in the susceptibility of *Drosophila* to infection [32]. For these reasons we wished to include immunity genes in our dataset. Because many immunity genes are only expressed at low levels in uninfected flies, we inoculated flies with heat-killed bacteria to upregulate immune response genes. For use in these inoculations, we prepared a cocktail containing gram-negative (*Escherichia coli*) and gram-positive (*Micrococcus luteus*) bacteria, which will activate the Toll and IMD pathways which are the main immune signaling pathways of *Drosophila* [33]. We grew overnight cultures of the two species separately in Luria broth at 37 °C with constant shaking and spun them down the following day at 2200 g for five minutes. To wash the pellets, we resuspended them in 500 ml Ringer’s solution and spun down as before. The wash was repeated three times and each mixture diluted until the optical density at 600 nm reached 1.0. We then heat killed the suspended bacteria at 80 °C for 30 min, plated 100 ul of each mixture, incubated at 37 °C, and checked for growth the next day. Upon observing no growth, we combined the two suspensions in equal proportions, aliquoted the mixture, and stored the aliquots at -80 °C.

We immune challenged 6–9 day-old female flies per line from the crosses described above by injecting them with 69 nl of the bacterial cocktail. We injected flies in the ventral anterior of the abdomen between 9 am and 2 pm, snap froze them in groups of 10 in liquid nitrogen 17–21 h post-injection, and stored them at -80 ° C. To avoid confounding results with a batch, time of day, or day effect, lines were divided into four blocks (A, B, C, D) containing approximately four lines from each population (16 lines per block). Injections were carried out on two blocks a day over the course of six days and blocks were staggered so that each was paired with every other and each appeared at either the beginning or end of the day.

### RNA extraction, library preparation, and sequencing

To extract RNA from frozen flies, we homogenized flies in groups of 10 in TRIzol and then used Direct-zol RNA MiniPrep kits according to the manufacturer’s protocol (Zymo Research). To remove residual DNA, we used TURBO DNA-free kits according to protocol (Invitrogen). We verified sample purity and integrity by measuring ratios of light absorbance at optical densities of 260 nm and 280 nm and using the Agilent 2000 Bioanalyzer. In all, 15, 16, 13, and 12 lines with high quality RNA were selected from Bowdoinham, Homestead, Melbourne and Innisfail respectively. For 4 lines per population, libraries were prepared for two biological replicates, and for the remaining lines, libraries were prepared for one biological replicate. Sets of replicates were taken from R1 and R2 of the cross, while non-replicated genotypes were taken solely from R2, except in three cases in which RNA extraction failed, and then these were taken from R1. Libraries were prepared by the Genome Analysis Centre (TGAC) (Norwich, UK) using the Illumina TruSeq RNA Sample Prep Kit v2. Libraries were pooled in groups of 14 or 15 samples, with populations evenly divided amongst lanes, and sequenced with 5 lanes of 100 bp paired-end reads on an Illumina HiSeq2000 at TGAC.

### Total gene expression analysis

Reads were aligned to the *D. melanogaster* reference sequence (Ensembl build BDGP5.25) using TopHat2 (version 2.0.8 with Bowtie2) while allowing 10 mismatches with parameters described in Quinn et al. [28]. The number of reads mapped per annotated protein-coding gene was enumerated using HTSeq [42], and differential gene expression analysis was performed using edgeR version 3.2.4 [36]. Principle component analysis revealed three clear outliers in total gene expression (1 line from Bowdoinham, 2 lines from Homestead), and these were excluded from subsequent analyses. Prior to differential gene expression analysis, reads were down-sampled to a fixed coverage level (6,968,765 reads per sample) by randomly sampling paired reads prior to alignment, and 10 samples were analysed per population without replication. Separately for the United States and Australia, we estimated differential expression between tropical and temperate populations. To filter out lowly expressed genes, we required that each gene have at least 1 count per million (cpm) in at least 10 samples to be retained. This resulted in 8717 genes being analysed in the United States and 8739 in Australia. EdgeR was also used to obtain per-gene estimates of cpm for each individual. Gene Ontology (GO) term enrichment was performed using the online analysis tool GOrilla [43]. In all cases, enrichment of GO term categories of significant genes was assessed by comparison against a background list of all genes which met our filtering criteria (*P*-value threshold of 0.001).

### Allele Specific Expression (ASE) estimates and analysis

The methods used to estimate ASE are briefly summarised here, as they have previously been described in detail by Quinn et al. [28], including an exploration of the performance of the methods on the sequences from one of the samples described here. To estimate ASE, reads were first aligned as described above, and non-uniquely mapped reads were discarded. To call SNPs use the software VarScan [44]. We then filtered the called SNPs by requiring them to have an increasing number of supporting reads (base quality greater than 19) as the depth of coverage increased. This threshold was set such that the probability of the number of errors exceeding this threshold was less than 0.0001 if every read had the lowest possible quality score and a systematic bias resulted in all errors causing the same incorrect nucleotide to be called (a phred score of 20, which equates to an error rate of 1 in 100) [28]. In addition to this we eliminated SNPs at positions with fewer than 15 total reads, SNPs with more than two states (i.e. alleles), and SNPs that were not in genes (those that did not intersect with the GTF transcript annotation file) [28]. Finally, SNPs not called homozygous in at least two *Drosophila* Genetic Reference Panel (DGRP Freeze 2) lines were removed [28].

SNPs that passed the filters were used to create an alternate reference sequence and the previous steps were repeated, but this time reads were aligned to the alternate reference [28]. This step avoids a bias towards mapping reads that are more similar to the reference genome [28]. Because one of our genomes is the published reference, this alternate reference is correctly phased and should contain SNPs found in the wild isofemale line [28]. Note that the reference genome strain carries multiple phenotypic markers that ensure it has not been contaminated. Finally, reads from both alignments were combined using a custom script to ensure no read was included twice, and the SNP calls were used to get the per-gene counts for reads mapping to reference and wild genomes. Where different SNPs assigned the same read to both the reference and non-reference allele, either the read or the SNP was removed following the approach of Quinn et al. [28].

We separately tested each protein-coding gene for ASE using a generalised mixed effects model (GLMM) implemented by maximum likelihood in R version 3.0.2. We only included genes where at least 12 samples had biological replicates. In the GLM the reference and wild read counts were treated as binomially distributed response variables using a logit link function using the R function *glmer*. The fly genotype was treated as a random effect. Overdispersion was accounted for by also including biological replicate as a random effect (i.e. a residual variance was estimated). We tested for ASE by dropping the random effect of fly genotype from the model and testing whether this was significant using a likelihood ratio test. False discovery rates were calculated using the Benjamini and Hochberg [45] method with the R function *p.adjust*. For each gene, we also estimated the mean proportion of reads mapping to the reference allele using the GLM to allow us to separate genes into those biased towards and away from the reference allele.

For each gene with ASE, we divided the lines into two groups, those with a high or a low expression allele, based on the ratio of reads mapping to the wild versus reference genome alleles (see Fig. 2). To do this we first sorted lines in order of the log_2_ ratio of reads coming from the wild vs. reference alleles (where read counts from biological replicates were summed together). We then divided this list of lines into two groups. The first group contained the lines with the top log_2_ ratios and ranged in size from 1 to n-1, where n was the number of non-replicated lines. The second group was composed of the remaining lines. For each grouping, we fitted the GLM described above with the addition of a fixed effect of Group. We then extracted the log likelihood scores from these models, and the best grouping was taken to be the one with the maximum likelihood. The group with the mean log_2_ wild/reference ratio closest to 0 (i.e. similar levels of expression of wild and reference alleles) was taken to be the reference CRE group, and the other group was the alternate CRE group. If the alternate group had a log_2_ wild/reference ratio greater than 0, the wild allele was assumed to have higher expression than the reference allele, otherwise it was assumed to be lower. Examples for groups fit using this method are given in Fig. 2. To obtain the effect of population on ASE, we extracted mean ASE for each population by including Population as a fixed effect in the model described in the previous paragraph. We tested for the significance of latitude by including Latitude, Continent and a Latitude X Continent interaction in the model described in the previous paragraph.

## Additional files

Additional file 1: Figure S1. Genes that are significantly latitudinally differentiated in ASE. **Figure S2.** Downsampling latitudinally differentiated genes. **Figure S3.** Mapping bias towards alleles matching the reference genome does not explain the observed patterns. **Figure S4.** Mapping bias does not explain the correlation between continents in latitudinal differences in ASE after accounting for allele frequencies. **Table S1.** Log_2_ fold differences in total expression between low and high latitude populations were measured separately for the United States and Australia. Log_2_ fold changes, *P*-values, FDR values, and mean counts per million (cpm) for both continents are given for each gene that met our filtering criteria. Values are given on separate sheets for all genes and for only genes considered to be latitudinally differentiated (FDR<0.20 in population and same direction of change in both populations). **Table S2.** Significance values for ASE are given for each gene included in the final analysis. Counts of reads mapping to the reference or wild alleles are given for all genes in which they were measured. **Table S3.** Individual chromosome arms and inversions are not enriched for genes latitudinally differentiated in total expression. **Table S4.** Individual chromosomal arms and inversions do not drive latitudinal correlation in ASE. (ZIP 9689 kb)
