# Supplementary material for: Latitudinal clines in gene expression and cis-regulatory element variation in Drosophila melanogaster
Source: BMC Genomics. 2016 Nov 28;17:981. doi: 10.1186/s12864-016-3333-7 (PMC5126864; doi:10.1186/s12864-016-3333-7)
Supplement: Additional file 1: Figure S1. — Genes that are significantly latitudinally differentiated in ASE. Figure S2. Downsampling latitudinally differentiated genes. Figure S3. Mapping bias towards alleles matching the reference genome does not explain the observed patterns. Figure S4. Mapping bias does not explain the correlation between continents in latitudinal differences in ASE after accounting for allele frequencies. Table S1. Log2 fold differences in total expression between low and high latitude populations were measured separately for the United States and Australia. Log2 fold changes, P-values, FDR values, and mean counts per million (cpm) for both continents are given for each gene that met our filtering criteria. Values are given on separate sheets for all genes and for only genes considered to be latitudinally differentiated (FDR<0.20 in population and same direction of change in both populations). Table S2. Significance values for ASE are given for each gene included in the final analysis. Counts of reads mapping to the reference or wild alleles are given for all genes in which they were measured. Table S3. Individual chromosome arms and inversions are not enriched for genes latitudinally differentiated in total expression. Table S4. Individual chromosomal arms and inversions do not drive latitudinal correlation in ASE. (ZIP 9689 kb) [file 12864_2016_3333_MOESM1_ESM.zip › Juneja_Supplementary Material.docx]

**Additional file 1**

**Supplementary Figure Legends**

**Figure S1. Genes that are significantly latitudinally differentiated in ASE. (A)** Genes with a significant effect of latitude on ASE are shown (*P*<0.05), after excluding genes with a significant interaction between latitude and continent (*P*<0.05). For each gene, mean log_2_ fold differences in total expression (TE) between tropical and temperate populations are shown separately for the United States (US) and Australia (AU). Mean log_2_ ratio of expression of the wild and reference alleles (ASE) is shown separately for each population. A ratio of 0 indicates equal expression of wild and reference alleles, a ratio greater than 0 indicates higher expression of the wild allele (which should increase total expression in the absence of trans effects), and a ratio of less than 0 indicates higher expression of the reference allele (which should decrease total expression). (B)

The gene encoding (6-4) photolyase, which repairs UV-induced DNA damage, has higher total expression in tropical populations, caused by an increased frequency of high expression CRE(s). The frequency of high and low expression CRE alleles is shown with pie charts, and the corresponding distribution of total expression (counts per million) in each population is shown in the lower panel.

**Figure S2. Downsampling latitudinally differentiated genes (**A) Latitudinally differentiated genes had slightly higher average depth of coverages, which could affect the power to detect ASE, (B) so were downsampled to similar depths of coverage as non-differentiated genes. (C) After downsampling with 1000 replicates, the proportion of latitudinally differentiated genes with significant ASE decreased slightly (compared with mean proportion prior to downsampling, red line), but did not overlap with the mean proportion observed in non-differentiated genes (black dotted line). The maximum *P*-value (Fisher’s Exact Test) observed for the difference was 1.61x10^-9^.

**Figure S3. Mapping bias towards alleles matching the reference genome does not explain the observed patterns.** The analyses described in (A, B) Figures 1b, (C, D) Figure 3a, (E,F) Figure 3b, (G, H) Figure 4a, and (I-L) Figure 4c were repeated for reference and wild biased genes. The analyses showed the same qualitative pattern described in the main text, except (H) where the correlation is diminished in wild biased genes. This can be explained by differences in allele frequencies between reference and wild biased genes (see Figure S4). Reference biased alleles were defined to have an estimated mean population frequency of greater than 55%, and wild biased genes had a frequency below 45%.

**Figure S4. Mapping bias does not explain the correlation between continents in latitudinal differences in ASE after accounting for allele frequencies.** The reference allele is expected to be more common in the population because of ascertainment bias (i.e. rare alleles are less likely to have been sampled in the reference genome). The analysis shown in Figure 4a was repeated for reference and wild biased genes with estimated allele frequencies of 0-10%, 10-20%, 20-30%, 30-40%, and 40-50%. At low population frequencies, there was little correlation between continents in ASE differences, whereas at high frequencies, reference and wild biased genes showed the same qualitative patterns.

**Supplementary Table Legends.**

**Table S1.** Log_2_ fold differences in total expression between low and high latitude populations were measured separately for the United States and Australia. Log_2_ fold changes, *P*-values, FDR values, and mean counts per million (cpm) for both continents are given for each gene that met our filtering criteria. Values are given on separate sheets for all genes and for only genes considered to be latitudinally differentiated (FDR<0.20 in population and same direction of change in both populations). **[attached]**

**Table S2.** Significance values for ASE are given for each gene included in the final analysis. Counts of reads mapping to the reference or wild alleles are given for all genes in which they were measured. **[attached]**

**Table S3. Individual chromosome arms and inversions are not enriched for genes latitudinally differentiated in total expression.** Latitudinally differentiated genes were homogeneously distributed amongst chromosome arms (Fisher’s Exact Test *P*=0.2844). No individual chromosome arms or inversions were enriched for latitudinally differentiated genes when compared with the rest of the genome (Fisher’s Exact Test). The positions of chromosome inversions were obtained from Corbett-Detig and Hartl 2012[7].

**Table S4. Individual chromosomal arms and inversions do not drive latitudinal correlation in ASE.** Individually excluding chromosomal arms or inversions does not remove the significance (Spearman’s rank correlation) of the correlation between continents in ASE differences between tropical and temperate populations (see Figure 4a). The positions of chromosome inversions were obtained from Corbett-Detig and Hartl 2012[7].


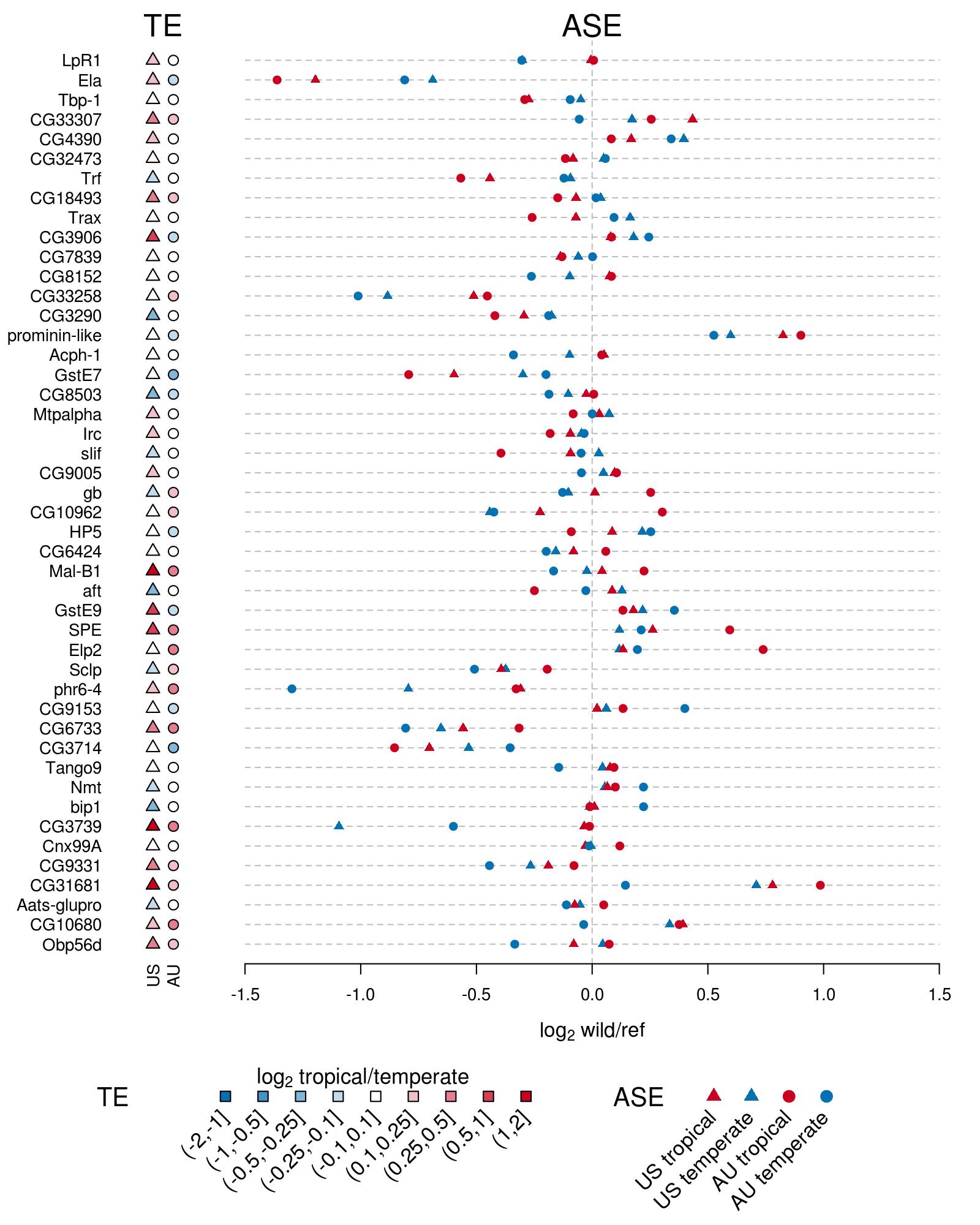


A


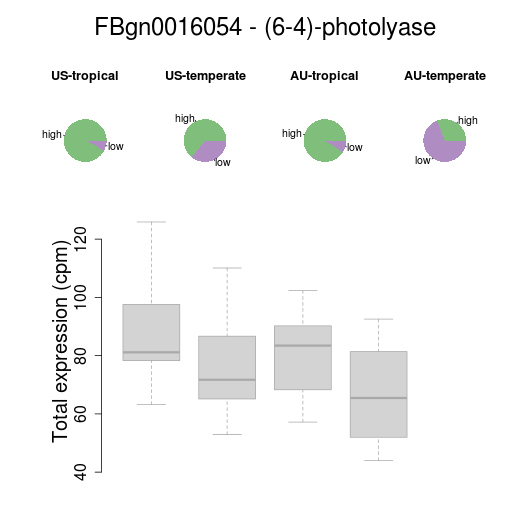


B

**Figure S1 (associated with Figure 3).**

**
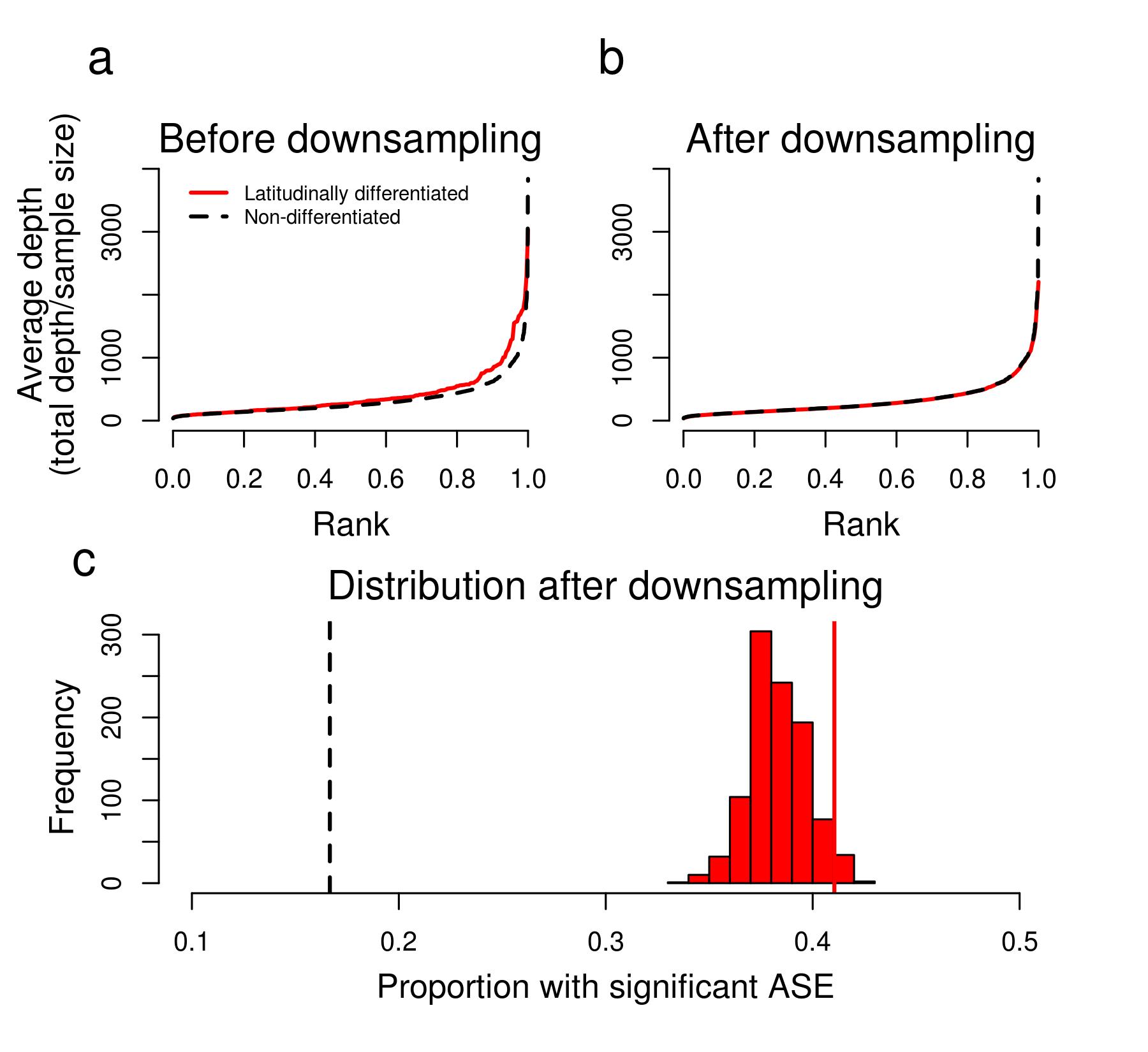
**

**Figure S2 (associated with Figure 4).**

**
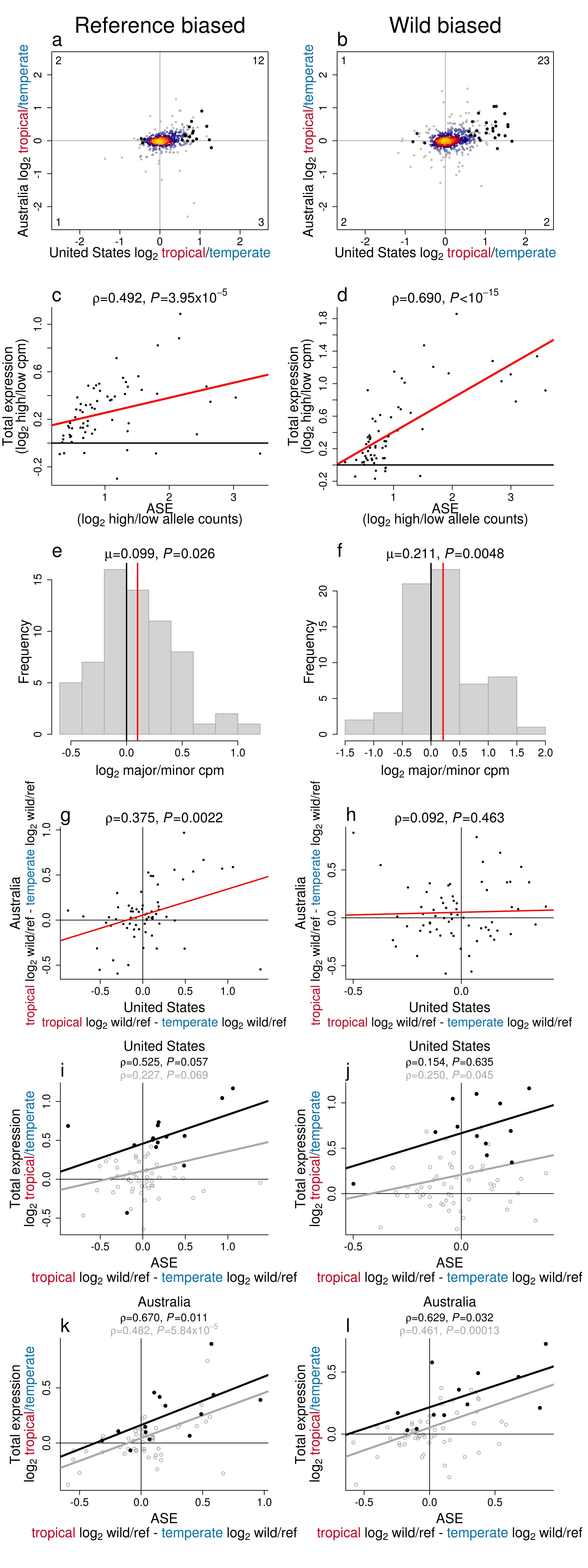
**

**Figure S3.**

**
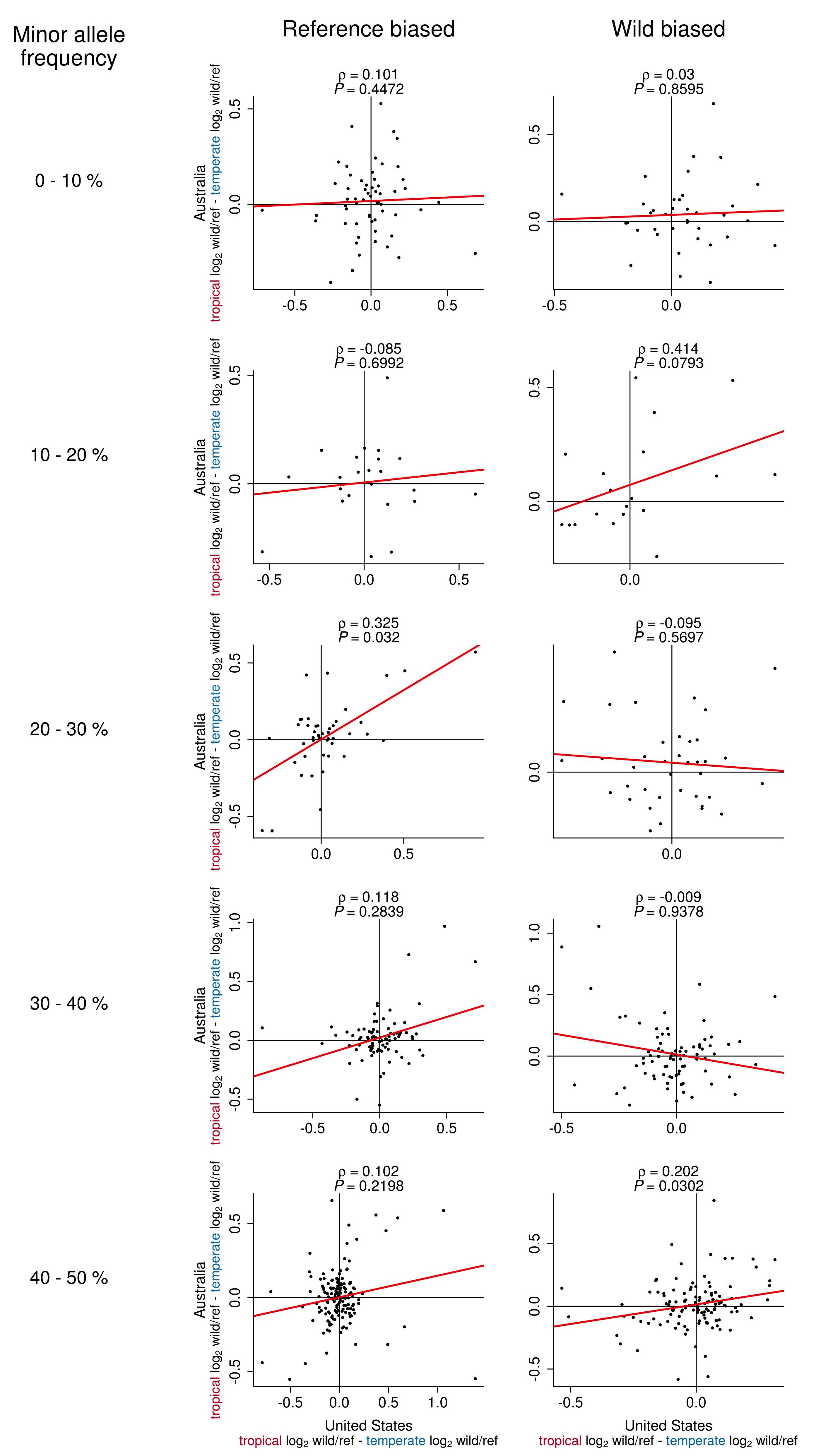
**

**Figure S4.**

**Supplementary Tables.**

**Tables S1 & S2. [attached]**

**Table S3.**

| **Chr. Arm** | **Latitudinally DE Genes** | **# of Genes Tested** | **%** | ***P*** |
| --- | --- | --- | --- | --- |
| 2R | 31 | 1807 | 1.72% | 0.7677 |
| 2L | 30 | 1591 | 1.89% | 0.8370 |
| 3R | 46 | 2163 | 2.13% | 0.2673 |
| 3L | 21 | 1643 | 1.28% | 0.0658 |
| X | 31 | 1437 | 2.16% | 0.3330 |
| **Total** | **159** | **8641** | **1.84%** |  |
|  |  |  |  |  |
| **Inversions** |  |  |  |  |
| In(2R)NS | 7 | 409 | 1.71% | 1.000 |
| In(2L)t | 14 | 898 | 1.56% | 0.6001 |
| In(3R)P | 11 | 633 | 1.74% | 1.000 |
| In(3R)K | 28 | 1155 | 2.42% | 0.1251 |
| In(3R)Mo | 13 | 570 | 2.28% | 0.4177 |
| In(3L)P | 14 | 895 | 1.56% | 0.6000 |

**Table S4.**

| **Excluded region** | **ρ** | ***P*-value** |
| --- | --- | --- |
| **2R**  **In(2R)NS^1^** | 0.163  0.145 | 0.0002  0.0003 |
| **2L**  **In(2L)t^1^** | 0.125  0.129 | 0.0050  0.0023 |
| **3R**  **In(3R)P^1^**  **In(3R)K^2^**  **In(3R)Mo^2^** | 0.082  0.115  0.101  0.122 | 0.0658  0.0045  0.0164  0.0026 |
| **3L**  **In(3L)P^1^** | 0.129  0.133 | 0.0037  0.0015 |
| **X** | 0.156 | 0.0012 |

^1^Common cosmopolitan inversion

^2^Rare cosmopolitan inversion

**References**

1. Adams, M. D. (2000). The Genome Sequence of *Drosophila melanogaster*. Science *287*, 2185–2195.

2. Quinn, A., Juneja, P., and Jiggins, F. M. (2014). Estimates of allele-specific expression in Drosophila with a single genome sequence and RNA-seq data. Bioinformatics *30*, 2603–10.

3. Anders, S., McCarthy, D. J., Chen, Y., Okoniewski, M., Smyth, G. K., Huber, W., and Robinson, M. D. (2013). Count-based differential expression analysis of RNA sequencing data using R and Bioconductor. Nat. Protoc. *8*, 1765–86.

4. Robinson, M. D., McCarthy, D. J., and Smyth, G. K. (2010). edgeR: a Bioconductor package for differential expression analysis of digital gene expression data. Bioinformatics *26*, 139–40.

5. Eden, E., Navon, R., Steinfeld, I., Lipson, D., and Yakhini, Z. (2009). GOrilla: a tool for discovery and visualization of enriched GO terms in ranked gene lists. BMC Bioinformatics *10*, 48.

6. Benjamini, Y., and Hochberg, Y. (1995). Controlling the False Discovery Rate: A Practical and Powerful Approach to Multiple Testing. J. R. Stat. Soc. Ser. B (Methodological). *57*, 289–300.

7. Corbett-Detig, R. B., and Hartl, D. L. (2012). Population genomics of inversion polymorphisms in Drosophila melanogaster. PLoS Genet. *8*, e1003056.
